# The performance of the MODY calculator in a non-Caucasian, mixed-race population diagnosed with diabetes mellitus before 35 years of age

**DOI:** 10.1186/s13098-023-00985-3

**Published:** 2023-02-06

**Authors:** Augusto Cezar Santomauro, Áurea Luiza Fernandes Magalhães, Flávia Tedesco Motta, Lucas Santos de Santana, Pedro Campos Franco, Silvia Maria de Freitas, Jeniffer Johana Duarte Sanchez, Aline Dantas Costa-Riquetto, Milena G. Teles

**Affiliations:** 1 Grupo de Diabetes Monogênico (Monogenic Diabetes Group), Unidade de Endocrinologia, Genética (LIM25), Unidade de Diabetes, Hospital das Clínicas, Faculdade de Medicina, Universidade de São Paulo (HCFMUSP), São Paulo, SP 01246-903 Brazil; 2 Department of Statistics and Applied Mathematics, Postgraduate Program in Modeling and Quantitative Methods, Science Center, Pici Campus, Federal University of Ceará (UFC), Fortaleza, CE 60440-900 Brazil

**Keywords:** MODY, MODY calculator, Type 1 diabetes mellitus, Type 2 diabetes mellitus

## Abstract

**Background:**

A maturity-onset diabetes of the young (MODY) calculator has been described and validated for use in European Caucasians. This study evaluated its performance in Brazilians diagnosed with diabetes mellitus (DM) before 35 years of age.

**Methods:**

The electronic records of 391 individuals were reviewed in 2020 at the diabetes clinic of a quaternary hospital in São Paulo were analyzed: 231 with type 1 DM (T1DM), 46 with type 2 (T2DM) and 114 with MODY. The MODY calculator was applied to the three groups. A receiver operating characteristic curve was calculated to obtain cut-off points for this population.

**Results:**

The principal differences between the MODY and the T1DM and T2DM groups were body mass index, a positive family history of diabetes and mean HbA1c level. Age at diagnosis in the MODY group was only significantly different compared to the T2DM group. Specificity and sensitivity were good for the cut-off points of 40%, 50% and 60%, with the accuracy of the model for any of these cut-off points being > 95%.

**Conclusion:**

The capacity of the calculator to identify Brazilian patients with MODY was good. Values ≥ 60% proved useful for selecting candidates for MODY genetic testing, with good sensitivity and specificity.

## Background

Maturity-onset diabetes of the young (MODY) is a rare, autosomal dominant, inherited disease that accounts for 1–4% of all cases of diabetes in individuals diagnosed before 35 years of age [1]. The differential diagnosis between the types of diabetes in this age group is challenging. The principal characteristics of MODY include, in addition to the early onset, the presence of a family history of 2–3 generations of diabetes diagnosed prior to 35 years of age, the presence of detectable C-peptide levels (> 0.6 ng/dl) 5 years after diagnosis of hyperglycemia and the absence of islet autoantibodies [1, 2]. The type of genetic testing most commonly indicated for conditions in which there are numerous genes involved in the possible etiology is massively parallel sequencing (multi-gene panel testing for the simultaneous evaluation of multiple genes) [1]. However, autoantibody measurement and genetic testing for MODY, which are expensive but could change the treatment and prognosis of the disease, are not always available [3].

In 2012, Shields et al. developed the MODY Probability Calculator, which analyzes clinical and laboratory-based characteristics to predict the probability of a diagnosis of MODY in individuals with diabetes mellitus (DM), thus screening and identifying candidates for genetic testing. Cut-off values of 10% and 25% resulted in sensitivity and specificity of over 85% compared to patients with type 1 DM (T1DM) and those with type 2 DM (T2DM) [4].

However, this tool has only been validated for use in a European Caucasian population. There are no data available for the mixed-race Brazilian population [5, 6] that would enable appropriate cut-off points to be defined for referring patients with a current diagnosis of T1DM or T2DM for genetic testing. Therefore, the objective of the present study was to evaluate the performance of the MODY calculator in Brazilians diagnosed with DM prior to 35 years of age.

## Methods

A retrospective analysis was performed of electronic medical records containing the clinical and laboratory-based data of male and female patients diagnosed with DM at 1 to 35 years of age. In 2020, patients who were being followed-up at part-time, twice-weekly diabetic outpatient clinics providing care exclusively to patients with T1DM and T2DM were retrospectively selected for inclusion in the study. These diabetes clinics are part of the Department of Endocrinology and Metabolic Diseases, Teaching Hospital, School of Medicine, University of São Paulo. The following inclusion criteria were applied: live patients being followed up at these clinics and who had been diagnosed with DM prior to 35 years of age. Most of these patients had been followed up for around 11 years, with one patient having been monitored for 67 years, since 1953. Patients receiving care at the outpatient clinic for individuals with a diagnosis of MODY were also selected using the following inclusion criteria: a diagnosis of DM prior to 35 years of age and genetic tests performed between 2012 and 2020 showing a pathogenic or probably pathogenic variant. A total of 528 patient records were reviewed. Of these, 137 did not meet the inclusion criteria. The remaining 391 patients were retrospectively evaluated: 231 with a diagnosis of T1DM, 46 with a diagnosis of T2DM and 114 with a diagnosis of MODY. In accordance with the methodology described in the study conducted by Shields et al. [4], patients who began insulin treatment less than six months after diagnosis were considered to have T1DM, while those who did not meet this criterion were classified as T2DM. A confirmed diagnosis of MODY was based on positive genetic testing using Sanger sequencing or massively parallel sequencing with a custom gene panel, as already described [5]. Genetic testing results were as follows: 1 patient with a mutation in the *HNF4A* gene, 80 in the *GCK* gene, 20 in the *HNF1A* gene, 2 in the *PDX1* gene, 6 in the *HNF1B* gene, 2 in the *NEUROD1* gene, 1 in the *INS* gene and 2 in the *ABCC8* gene [7, 8]. Only pathogenic and likely pathogenic variants were included, as defined by the American College of Medical Genetics and Genomics and the Association for Molecular Pathology (ACMG/AMP) [9].

The MODY probability model was applied using the smartphone application “Exeter Diabetes App”, developed by the University of Exeter in the United Kingdom [10]. The calculator assesses the following parameters: age at diagnosis of diabetes, current age, sex, ethnicity, body mass index (BMI), parental history of DM, glycated hemoglobin (HbA1c) at diagnosis, current use of oral anti-diabetes medication, insulin therapy and time of starting it, and the presence of certain associated clinical characteristics such as renal cysts, deafness, partial lipodystrophy, and severe insulin resistance in the absence of obesity or severe obesity, together with other syndromic features. Ethnicity was dichotomized into Caucasian or non-Caucasian based on the individual’s self-reported skin color.

### Statistical analysis

In the descriptive analysis, the continuous variables were expressed as measures of central tendency (medians and interquartile ranges [IQR]), and the categorical variables as percentages. Since the distribution of the continuous variables was not normal (Anderson–Darling test), the non-parametric Mann–Whitney test and the Brunner-Munzel test were used, as the variables were homogenous and heterogenous, respectively (Bartlett’s test). For the categorical variables, the chi-square test and Fisher’s exact test were used. The variables were described using boxplots, bar charts and frequency graphs. The receiver operating characteristic (ROC) curve was used to obtain cut-off points for the calculator in order to determine the most appropriate value for differentiating between patients with MODY and those with T1DM or T2DM. Measures of sensitivity and specificity were calculated for the different cut-off points, beginning at 10% and ending at 70%. The R software program, version 4.1.3, was used throughout the statistical analysis. The significance level adopted in the tests was 0.05.

## Results

**Fig. 1:**
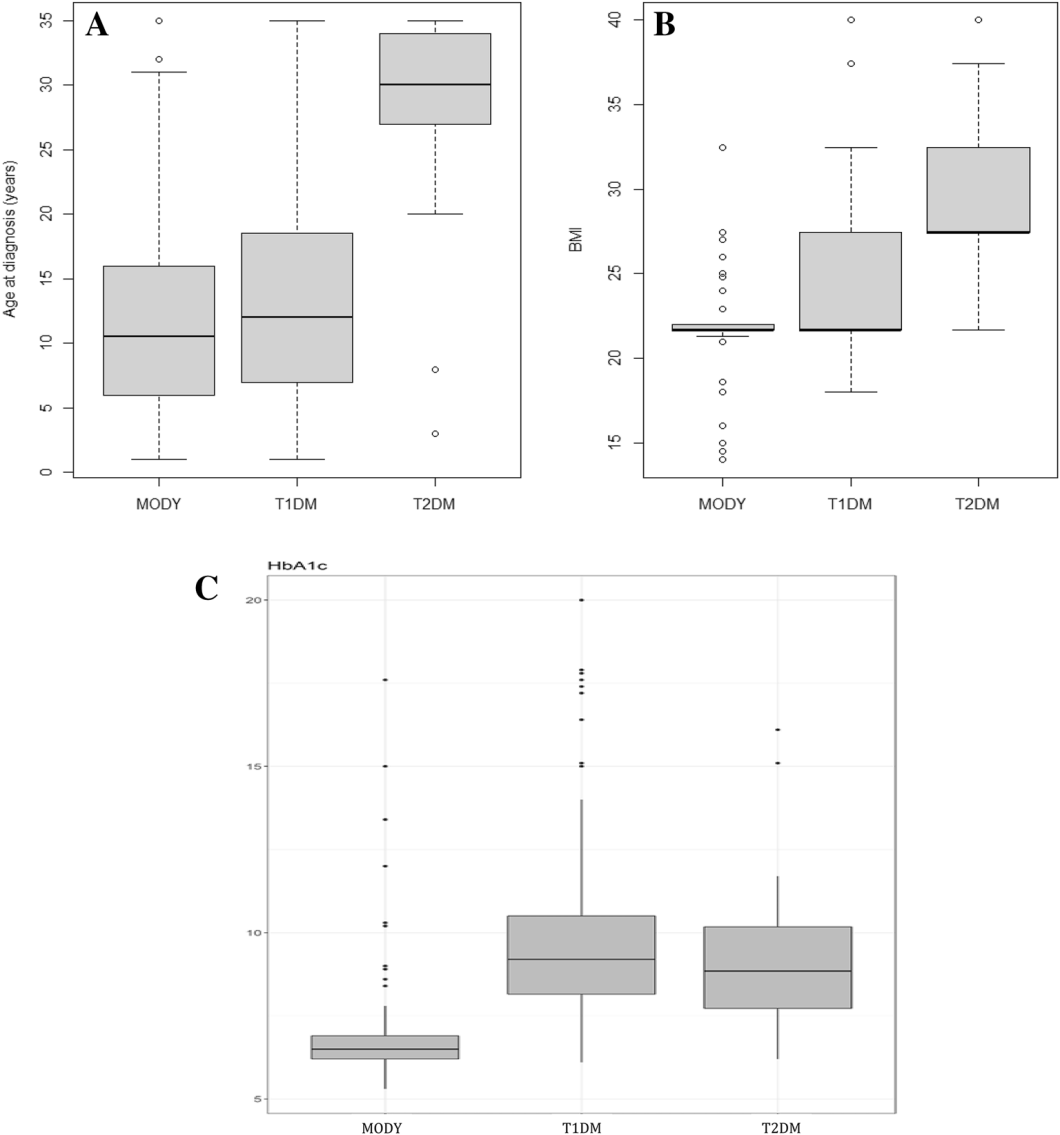
Comparison in the groups of patients with maturity-onset diabetes of the young (MODY), type 1 diabetes mellitus (T1DM) and type 2 diabetes mellitus (T2DM). **A** Age at diagnosis. **B** Body mass index (BMI; kg/m^2^). **C** HbA1c (%)

The median age at diagnosis in this cohort was 10.5 years (IQR: 6–16 years) for the MODY group, 12 years (7–18.5 years) for the T1DM group and 30 years (27.3–34 years) for the T2DM group (MODY vs. T1DM: p = 0.136; MODY vs. T2DM: p < 0.001) (Fig. 1A). In the MODY group, 47.4% of the patients were male compared to 37.2% in the T1DM group and 47.8% in the T2DM group, with no statistically significant difference between the groups. All the patients in the MODY group self-reported as non-white compared to 12.55% of the T1DM group and 19.57% of the T2DM group. In the MODY group, 74.56% of the participants had a normal BMI compared to 49.35% in the T1DM group and 15.22% in the T2DM group (p < 0.001).

**Fig. 2:**
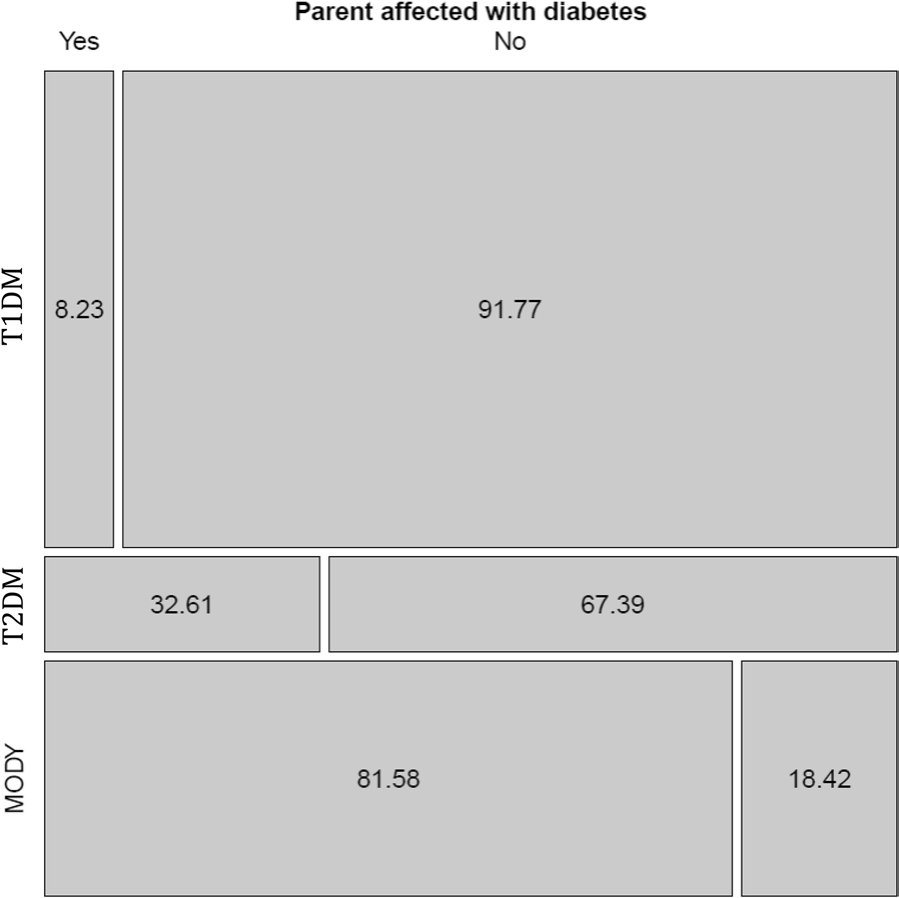
Comparison between the percentage of individuals with a family history in the groups of patients with maturity-onset diabetes of the young (MODY), type 1 diabetes mellitus (T1DM) and type 2 diabetes mellitus (T2DM)

A greater proportion of patients in the T2DM group were overweight or obese compared to the MODY and T1DM groups (84.79% vs. 10.53% and 47.62%, respectively). The median BMI of the patients diagnosed with MODY was 21.71 kg/m^2^ compared also to 21.7 kg/m^2^ for those with T1DM and 27.5 kg/m^2^ for those with T2DM, with this difference being statistically significant (p < 0.001) (Fig. 1B). Of the participants with a diagnosis of MODY, 81.58% had a positive family history of diabetes compared to 32.61% of the participants with T2DM and only 8.23% of those with T1DM (p < 0.001) (Fig. 2). In relation to the time until the initiation of insulin therapy, only 3.51% of the patients with MODY began using insulin immediately following diagnosis, with the majority (86.6%) not currently using insulin. The median time of follow-up of these MODY patients was 7 years (IQR: 7–17 years).

The median initial HbA1c measurement available, preferably that performed closest to diagnosis, in the MODY, T1DM and T2DM groups was 6.5% (IQR: 6.2–6.9%), 9.2% (8.2–10.5%) and 8.9% (7.7–10.2%), respectively, (p < 0.001) (Fig. 1C).

**Fig. 3:**
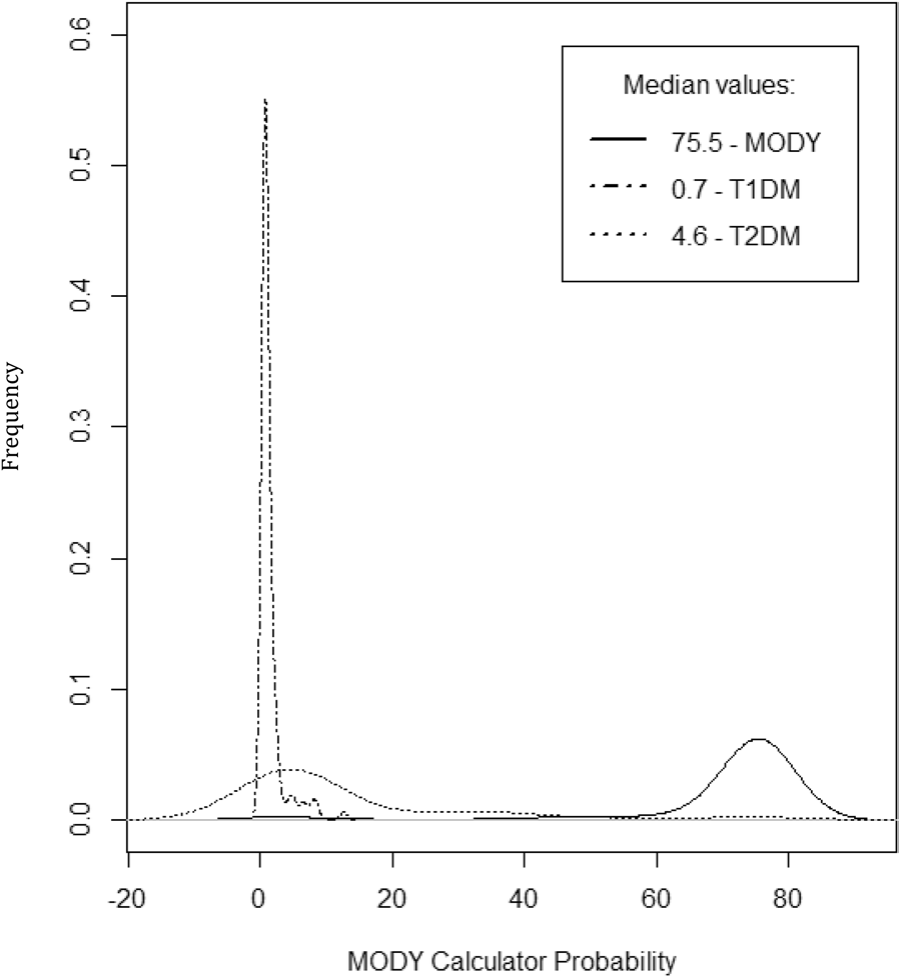
Comparison of the probability calculator in the group of patients with maturity-onset diabetes of the young (MODY), type 1 diabetes mellitus (T1DM) and type 2 diabetes mellitus (T2DM)

The median probability of the MODY calculator was 75.5% for MODY, 0.7% for T1DM and 4.6% for T2DM, with the inter-group comparisons being statistically significant (p < 0.001) (Fig. 3).

**Fig. 4:**
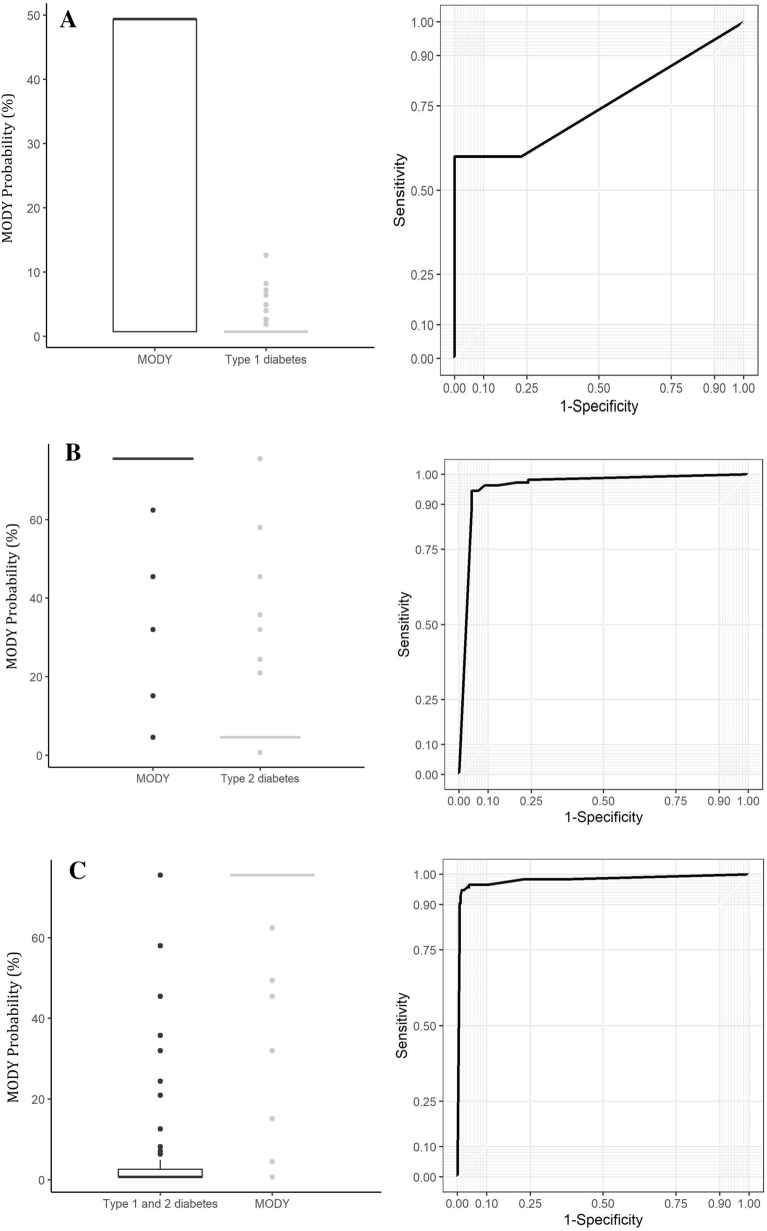
**A** Graph of the receiver operating characteristic (ROC) curve for patients with maturity-onset diabetes of the young (MODY) who initiated insulin treatment less than six months after diagnosis versus type 1 diabetes mellitus (T1DM). **B** Graph of the receiver operating characteristic (ROC) curve for patients with maturity-onset diabetes of the young (MODY) who did not initiate insulin treatment less than six months after diagnosis versus type 2 diabetes mellitus (T2DM). **C** Graph of the receiver operating characteristic (ROC) curve for all patients with maturity-onset diabetes of the young (MODY) versus type 1 diabetes mellitus (T1DM) versus type 2 diabetes mellitus (T2DM)

The cut-off points of 40%, 50% and 60% resulted in a good balance between sensitivity and specificity, with the accuracy of the model for any one of these cut-off points being above 95%. The criterion of maximum specificity with 95% sensitivity was best in relation to the desired cut-off level. According to the ROC curve and the area under the ROC curve (AUC), the goodness-of-fit of the model was satisfactory. Both in the comparison of the MODY group with the T1DM group and with the T2DM group, the cut-off point of the MODY calculator with maximum specificity and 95% sensitivity was 46%. If maximum sensitivity is used with 95% specificity, the cut-off point of the MODY calculator was 62% in the comparison with T2DM and 6% in the comparison with T1DM (Table 1, Figs. 4A and B).

**Table 1:** Different cut-off points of the maturity-onset diabetes of the young (MODY) calculator, comparing MODY with type 1 diabetes mellitus (T1DM) and type 2 diabetes mellitus (T2DM), with their respective specificity and sensitivity

Model	Cut-off for a probability of classification of MODY based on the logistic regression model (%)								
	10%	20%	30%	40%	50%	60%	70%	80%	90%
T1DM and T2DM versus MODY									
Sensitivity (%)	98.51	98.15	98.17	97.85	96.14	96.15	94.5	70.84	
Specificity (%)	89.43	90.83	92.37	96.43	97.17	98.1	98		
LR + for MODY (95%CI)	9.3203 (5.5729–15.5876)	10.7078 (6.0953–18.8107)	12.871 (6.8679–24.1213)	27.3979 (10.4645–71.7326)	33.9696 (11.1314–103.6652)	50.4808 (12.7912–199.2239)	47.2509 (11.9797–186.3684)	–	
PPV for MODY	78.27%	80.54%	83.26%	91.37%	92.92%	95.12%	94.81%	–	
LR− for MODY (95%CI)	0.0167 (0.0063 –0.0442)	0.0203 (0.0085–0.0485)	0.0198 (0.0083–0.0473)	0.0223 (0.0101–0.0493)	0.0397 (0.0222–0.0710)	0.0392 (0.0220–0.0700)	0.0561 (0.0348–0.0904)	–	
NPV for MODY	99.36%	99.22%	99.24%	99.15%	98.49%	98.51%	97.88%		
Accuracy	91.96%	92.87%	93.99%	96.82%	96.88%	97.55%	97.02%		

*LR* + positive likelihood ratio, *PPV* positive predictive value, *LR− *negative likelihood ratio, *NPV* negative predictive value

The boxplot in Fig. 4C compares the T1DM and T2DM patients as a single group with the MODY patients, and the differentiation between the former two groups and the latter group is clear. The ROC curve also shows the good capacity of the model to differentiate between the groups.

## Discussion

The present study successfully validated the performance of the MODY calculator in differentiating a large sample of patients with a confirmed diagnosis of MODY from patients with T1DM and T2DM and also succeeded in establishing a cut-off point for the probability of this diagnosis.

The originality of the present study lies in the application and validation of the MODY probability calculator in a large cohort of patients diagnosed with diabetes at an early age and who, furthermore, were not exclusively Caucasian but were of mixed race and of different ethnic origin, characteristics that make the Brazilian people a unique population worldwide [5, 6]. This study will allow the MODY calculator to be used within the country as a tool for estimating the probability of MODY prior to testing, thus screening and identifying individuals to be referred for genetic testing. This is particularly relevant since the frequency of MODY in the Brazilian population is uncertain and the diagnosis of this DM subtype is challenging [1].

Few studies involving patients with MODY have included non-Caucasian populations. Of note, Misra et al. [11] analyzed a population referred for genetic testing for MODY and compared the characteristics of the individuals of South Asian descent with those of Caucasians. The characteristics of South Asians with a diagnosis of MODY were found to be largely similar to those of the Caucasian patients with MODY, except for the fact that the South Asians had lower BMI and were younger at the time of diagnosis. The application of the MODY calculator could therefore be discriminatory in this population; however, the tool has yet to be validated for use in that ethnic group.

Non-Caucasian patients were also evaluated in a study conducted in Australia [12]. The prevalence of MODY and permanent neonatal diabetes mellitus was investigated in 1668 European and non-European patients with a diagnosis of T1DM or T2DM. Of those patients, 196 were under 35 years of age, 148 were of European descent, and, according to the probability calculator, 8% had a high probability of MODY. After genetic testing in these individuals, a diagnosis of MODY was confirmed in 3 patients. However, in the non-European group of patients (n = 46), although 28% had a high probability of MODY according to the calculator, none had the diagnosis confirmed at genetic testing. For the patients clinically diagnosed with T1DM or T2DM, the cut-off value used for referring patients for genetic testing for MODY was 25% with the use of the calculator. The prevalence of MODY in the participants of European descent was 0.28% (95%CI 0.09–0.77%) compared to 0 in the participants of non-European descent. This lower prevalence in that population could be due to the small number of individuals in that study, which could have limited the evaluation of the calculator. Furthermore, the cut-off point used for genetic testing was low, since it was based on the study conducted by Shields et al. [4], which suggests a cut off limit of between 10 and 25%.

In the present study, the principal factors distinguishing the MODY group from the T1DM and T2DM groups were BMI, a positive family history of diabetes and mean HbA1c value, with age at diagnosis only being a distinguishing factor when the MODY group was compared to the T2DM group.

The majority (85%) of the young people with T2DM had a BMI > 25 kg/m^2^ and this was an important distinguishing factor in the MODY patients, the majority (75%) of whom had a BMI within the normal range. Although approximately 35% of the patients in the present study with a diagnosis of T1DM were also overweight, there were few cases of obesity compared to the T2DM group.

In relation to family history, the finding that in 18.43% of MODY cases there was no family history of diabetes is intriguing. Our principal hypothesis for this finding is the absence of confirmed genetic testing in the parents or, in a minority of cases, the presence of a de novo mutation, as reported by Shields et al. [4] and in our recent description of a MODY cohort, which showed 24% of de novo mutations in GCK-MODY patients [13].

Most of the MODY patients were not in use of oral anti-diabetic drugs. This could be explained by the fact that this cohort consists primarily of individuals with GCK-MODY (70%) who typically show non-progressive mild hyperglycemia with no need for pharmacological treatment [2, 3, 13]. This characteristic of the present cohort was also reflected in the median HbA1c level of the MODY patients, which was significantly lower compared to that of the T1DM and T2DM groups.

Age at diagnosis was similar in the MODY group and the T1DM group. Therefore, as suggested by Shields et al. [4], completing the diagnostic investigation by measuring C-peptide and the main islet autoantibodies can be useful in distinguishing between MODY and T1DM, particularly in patients with an early diagnosis who are thin and have been in use of insulin therapy since diagnosis. A typical individual with T1DM could be diagnosed at a time when pancreatic beta-cell reserve is not yet depleted, and that individual may present with measurable C-peptide levels over the first five years of the disease. Likewise, positivity of the pancreatic antibodies falls as the disease progresses in those with a diagnosis of T1DM. In this institute, to avoid diagnostic confusion with T1DM, individuals with the following characteristics are referred for genetic testing: a diagnosis of DM prior to 25 years of age, C-peptide > 0.6 ng/dl for five years after diagnosis of DM and negative islet autoantibodies (anti-glutamic acid decarboxylase [anti-GAD], anti-insulin antibodies and anti-tyrosine phosphatase antibodies).

The present study included individuals with subtypes of MODY in addition to *HNF1A* and *GCK*, which are the most prevalent. The study conducted by Shields et al. [4] included only European Caucasians of 1 to 35 years of age who had a confirmed genetic diagnosis of HNF1A, HNF4A or GCK-MODY. In the present study, the population recruited was in the same age range; however, patients with a confirmed molecular diagnosis not only of *HNF4A*, *GCK* and *HNF1A* but also *PDX1*, *HNF1B*, *NEUROD1*, *INS* and *ABCC8* variants were included [5, 6]. All the variants included were pathogenic or likely pathogenic according to the ACMG/AMP criteria, with our group having experience in distinguishing between each one of these.

In relation to the performance of the MODY probability calculator in the patients with T1DM, T2DM and MODY, Fig. 4 shows the graphs generated according to the model used in the paper published by Shields et al. [4]. The differentiation between the groups, as shown in the ROC curve, depends to a certain extent on the size of the groups between which differentiation is to be performed. To render the group homogenous, in Fig. 4A the individuals who used insulin within six months of diagnosis were compared, i.e. those classified as T1DM according to the criteria used in the article published by Shields et al. [4]. Following these criteria, only five individuals had a diagnosis of MODY, since the majority of patients with MODY are known not to use insulin shortly after diagnosis [1]. Therefore, comparing these five individuals with the 231 individuals with a diagnosis of T1DM rendered the performance of the calculator less effective in differentiating between these two groups. Figure 4B, on the other hand, compares the individuals who began using insulin more than six months after diagnosis, including the patients classified as T2DM according to the criteria published by Shields et al. [4], and the majority of the patients with MODY. This comparison resulted in a ROC curve that reflected better differentiation between MODY and T2DM. It appears that in this mixed-race population, the performance of the MODY probability calculator is not as satisfactory when the individual initiates insulin therapy shortly after diagnosis. i.e. by comparing patients with a greater probability of being diagnosed with T1DM; however, its performance is adequate when differentiating patients with a greater likelihood of being diagnosed with T2DM. For this reason, Fig. 4C was then constructed. This figure compares T1DM and T2DM with MODY, attempting to mimic conditions in actual clinical practice where most individuals with MODY will not use insulin shortly after diagnosis. Therefore, the values of the probability calculator for differentiating individuals with MODY in this mixed-race population ended up being higher (around 60%) than the values published by Shields et al. [4], reflecting satisfactory differentiation with the patients with T2DM who also did not use insulin in the first six months after diagnosis.

The cut-off point of the MODY calculator that resulted in the best sensitivity and specificity in the present study was 60% (sensitivity 96.1% and specificity 98.1%), followed by 40% (sensitivity 97.8% and specificity 96.4%), with accuracy being over 95% for all cut-off values above 40%. These values exceed that used in the Australian study [12] (cut-off of 25%) and that suggested by Shields et al. [4] (cut-off of 25% when comparing MODY with T2DM and 10% when comparing MODY with T1DM); however, in the present mixed-race population, the accuracy of these cut-off points was even higher.

A previous Brazilian study [14] conducted in Rio de Janeiro evaluated the prevalence of GCK*-* and HNF1A*-*MODY in an ethnically diverse population with suggestive clinical characteristics and analyzed what the impact would be of using the probability calculator for this purpose. Thirty-four patients aged ≤ 35 years at diagnosis of diabetes, with BMI < 30, negative anti-GAD and anti-IA2 antibodies, and a positive family history of diabetes in at least two generations, were analyzed. Patients with T1DM, a history of diabetic ketoacidosis, clinical signs of insulin resistance and DM of secondary causes were excluded from the analysis. In that study, cut-off points for the probability of MODY > 75% and > 62% were found for patients with HNF1A-MODY and GCK-MODY, respectively. Those findings suggest that higher cut-off points should be considered as indicative of a need to screen for MODY in non-Caucasian and mixed-race populations such as that of Brazil. Nonetheless, that study was limited by its small sample size, the absence of a control group, the fact that C-peptide was not measured and that genetic testing was only performed for two MODY genes; moreover, using only Sanger sequencing. Although the cut-off points were relatively higher (> 75% for *HNF1A* and 62% for *GCK*) compared to those found in the present study, comparison was not made with individuals with T1DM or T2DM as control groups; therefore, at that moment, the effectiveness of the MODY calculator could not be validated or evaluated in this population.

In an attempt to differentiate between individuals with MODY and individuals with T1DM and T2DM, in addition to the probability calculator described by Shields et al. [4], other alternatives include the use of biomarkers. In 2012, Mughal et al. [15] described the use of apolipoprotein M (apoM) to differentiate individuals with HNF1A-MODY from those with T1DM. Lower values were found in patients with HNF1A-MODY, with an area under the ROC curve of 0.91. Other biomarkers such as high-sensitivity C-reactive protein (hs-CRP) have already been fully investigated to differentiate HNF1A-MODY from other types of DM [16] and still others appear promising, including 1,5-anhydroglucitol, which was able to successfully differentiate individuals with HNF1A-MODY from those with T2DM who had A1C of between 6.5 and 9.0% [17]. Despite the obvious usefulness of these biomarkers, it is important to remember that most of the studies in the literature evaluated only patients with HNF1A-MODY in which the clinical course of the condition is closer to that of individuals with T2DM. Furthermore, the cost and availability of these biomarkers can render their use in clinical practice unviable. Clinical characteristics are habitually used as differentiating factors in individuals with suspected T2DM prior to 35 years of age. These include signs of insulin resistance (abdominal obesity, acanthosis nigricans, hypertension or dyslipidemia) as well as the absence of autoantibodies and evidence of endogenous insulin secretion as shown by C-peptide levels [1]. Nevertheless, in the present study, we decided to adopt the classification used in the article published by Shields et al., who consider only the initiation of insulin therapy within six months of diagnosis as the differentiating factor between T1DM and T2DM in individuals under 35 years of age. We believe that, in doing so, the validation of the probability calculator in this mixed-race population would be closer to that of the original article. The probability calculator described by Shields et al. uses clinical, epidemiological and treatment data, making it a more practical and cost-effective tool for use in clinical practice. For this reason, biomarkers were not used in its construction.

In view of these findings and considering the cost of molecular genetic testing, we would suggest using a cut-off point of 60% for the Brazilian population. Bearing in mind the socioeconomic differences in the different regions of Brazil, in those regions where testing is more economically viable and more accessible to the population, a cut-off limit of 40% could be used.

The limitations of the present study include the small number of patients with a diagnosis of T2DM and not having measured C-peptide or islet autoantibodies to define T1DM. In addition, the patients with T1DM and T2DM did not undergo genetic testing to exclude the possibility of MODY; therefore, we cannot affirm that there are no MODY patients classified as T1DM or T2DM in this study. A study involving a larger group of patients and with genetic testing of all the participants could be useful for identifying the most effective cut-off point for these groups.

## Conclusion

The MODY calculator performed well at identifying patients with a diagnosis of MODY in the Brazilian population. In addition, it was able to differentiate this rare subtype from the more common types. Values ≥ 60% could be useful as a screening tool for referring individuals under 35 years of age with DM for genetic testing for MODY, with good sensitivity and specificity.

## Data Availability

The datasets used and/or analyzed during the current study are available from the corresponding author upon reasonable request.

## Abbreviations

MODY: Maturity-onset diabetes of the young
DM: Diabetes mellitus
T1DM: Type 1 diabetes mellitus
T2DM: Type 2 diabetes mellitus
ACMG/AMP: American college of medical genetics and genomics and the association for molecular pathology
BMI: Body mass index
IQR: Interquartile ranges
ROC: Receiver operating characteristic
AUC: Area under the curve
HbA1c: Glycated hemoglobin
anti-GAD: Anti-glutamic acid decarboxylase
apoM: Apolipoprotein M
hs-CRP: High-sensitivity C-reactive protein
1,5-AG: 1,5-Anhydroglucitol
